# Design and Study of Nanoceria Modified by 5-Fluorouracil for Gel and Polymer Dermal Film Preparation

**DOI:** 10.3390/ph16081082

**Published:** 2023-07-29

**Authors:** Nina Melnikova, Ilya Sheferov, Dmitry Panteleev, Anastasia Emasheva, Irina Druzhkova, Nadezhda Ignatova, Tatiana Mishchenko, Maria Vedunova

**Affiliations:** 1 Faculty of Chemistry, Lobachevsky State University, 23 Gagarin Av., 603022 Nizhny Novgorod, Russia; 2 Department of Pharmaceutical Chemistry, Privolzhsky Research Medical University, 10/1 Minin Sq., 603950 Nizhny Novgorod, Russia; panteleev_da@mail.ru; 3 Research Institute of Experimental Oncology and Biomedical Technologies, Privolzhsky Research Medical University, 10/1 Minin Sq., 603950 Nizhny Novgorod, Russia; 4 Institute of Biology and Biomedicine, Lobachevsky State University, 23 Gagarin Av., 603022 Nizhny Novgorod, Russia

**Keywords:** nanoceria modified by 5FU, polymer dermal films, hydrogel, cytotoxicity

## Abstract

In this work we studied nanoceria (CeO_2_NPs) and nanoceria modified by 5-fluorouracil (5FU) as potential APIs. Nanoceria were synthesized by precipitation in a matrix of hydroxyethyl cellulose or hydroxypropylmethyl cellulose, using cerium (III) nitrate and meglumine. Nanoceria properties were estimated by UV, FTIR and X-ray photoelectron spectra; scanning electron and atomic force microscopy; powder X-ray diffraction patterns and energy dispersive X-ray microanalysis. The cytotoxicity of nanoceria and polymer-protected nanoparticles was evaluated using the established cell line NCTC clone 929 (C3H/An mouse, subcutaneous connective tissue, clone of L. line). The morphology and metabolic activity of nanoparticles at 10 μg∙mL^−1^ of cells was not significant. In addition, the cytotoxic effects of nanoceria were assessed on two human colorectal cancer cell lines (HT29 and HCT116), murine melanoma B16 cells and normal human skin fibroblasts. An inhibitory effect was shown for HCT116 human colorectal cancer cells. The IC_50_ values for pure CeO_2_NPs and CeO_2_NPs-5FU were 219.0 ± 45.6 μg∙mL^−1^ and 89.2 ± 14.0 μg∙mL^−1^, respectively. On the other hand, the IC_50_ of 5FU in the combination of CeO_2_NPs-5FU was 2-fold higher than that of pure 5FU, amounting to 5.0 nmol∙mL^−1^. New compositions of nanoceria modified by 5-fluorouracil in a polymer matrix were designed as a dermal polymer film and gel. The permeability of the components was studied using a Franz cell.

## 1. Introduction

The cytostatic 5-fluorouracil (5FU) synthesized in 1957 by Heidelberger et al. [1] remains a very important drug due to its powerful pharmacological action, both systemically and locally [2,3]. The systemic use of 5FU leads to many undesirable side effects–inflammation, allergic reactions, stomatitis and esophago-pharyngitis (which can result in peeling and ulceration), diarrhea, anorexia, vomiting, etc. At the same time, oral absorption of 5FU is unpredictable and incomplete. For this reason, topical application of 5FU is of great interest. The application of 5-fluorouracil in various topical dosage forms has proven its value in the treatment of various skin diseases, such as actinic keratosis, multiple and superficial basal cell carcinomas and Bowen’s disease, as well as warts, psoriasis, vitiligo and melanoma [4,5,6,7,8,9,10,11].

The problem of the widespread use of 5FU in medical practice for topical application is its extremely low bioavailability associated with the high polarity of the molecule and low affinity for cell membranes [2]. Accordingly, when applied externally, 5FU has poor penetration and permeability in deep skin layers and requires large doses that cause cancer cell resistance and increased side effects (redness, itching, peeling, pain and burning during treatment, skin erosion) [12].

New carriers of 5FU are mainly nano-sized, which increases their affinity for the skin, and they can reduce toxicity and improve 5FU bioavailability [3,4,13,14,15,16,17,18,19,20,21,22,23]. The novel nanocomposite hydrogels were developed using 5FU loaded onto the reduced graphene oxide as a nanodrug. Then, this nanodrug was loaded into a polymeric matrix of functionalized arabinoxylan [16]. These nanocomposite hydrogels have proven to be pH-sensitive under different pH conditions for their sustained and controlled release. The influence of graphene oxide conjugated with a biopolymer on anticancer activity was estimated for a sodium alginate–graphene oxide composite hydrogel in the absence of 5FU [17]. Chitosan–gelatin hydrogels containing 5FU–alginate nanoparticles are thermosensitive [23]. Nanoparticles based on cyclodextrin polymers and cholesterol inclusion complexes loaded with 5FU and methotrexate could be applied as injectable vehicles [19].

The use of various polymers as 5FU carriers is implemented in dermal delivery systems, which is a good alternative to using nanosized carriers due to the possibility of combining various drugs, the ability to penetrate into deeper layers, the potential for dosage adjustment, and the economy of the production process [3]. The polymeric component allows for percutaneous transport of the drug into the systemic circulation [24,25,26]. Biodegradable natural or modified natural materials such as polysaccharides (cellulose and its derivatives, chitosan, alginates, etc.) are a convenient polymer matrix for 5FU immobilization. Polysaccharides also contribute to the development of drugs that can both improve 5FU lipophilicity and also reduce the dose, and they can act as a 5FU carrier [24,25,26,27,28]. Moreover, homogeneous polysaccharides show the protective effect on the intestinal mucositis induced by 5FU [29].

Cellulose and cellulose-based materials are both earth abundant and biocompatible, and they are suitable for the formation of inorganic nanostructures with metal oxide nanoparticles [30,31]. For example, compared with 5FU alone, magnetic Fe_3_O_4_ nanoparticles immobilized on rice straw cellulose as a 5FU carrier caused targeted action and a higher anticancer effect against colorectal cancer [32].

Nanoceria (CeO_2_NPs) immobilized on cellulose derivatives like Fe_3_O_4_ nanoparticles immobilized on rice straw cellulose can be an alternative carrier for 5FU. Nanocomposites of nanoceria with cellulose derivatives or their composites with polysaccharides were demonstrated to have an effect on stem cell proliferation scaffolds as well as having antioxidant, antibacterial and enzymatic mimetic activities [33,34,35,36,37,38,39,40,41,42]. Nanoceria effects are due to unique physicochemical properties of CeO_2_NPs [43]. Cerium in nanoceria can exist either in a reduced (Ce^+3^) or completely oxidized (Ce^+4^) state since it has partially filled 4f and 5d subshells. The relative ratio of Ce^3+^ to Ce^4+^ cerium ions in nanoceria depends on the particle size and pH [43]. Although nanoceria are the mixture of Ce_2_O_3_ and CeO_2_ with a non-stoichiometric nature, in the present study, we use the term CeO_2_NPs, as this is customary in the literature on nanoceria [36,37,38,40]. Nanoceria containing a high concentration of Ce^3+^ are of the greatest interest for medical use, including for cancer, and may be considered a promising drug to treat tumors [41,44,45]. The effect of cerium ions on the regeneration of integumentary tissues is well studied [41]. Thus, cerium nitrate accelerates the recovery of fibroblasts in the scab zone and during wound epithelization. A collagen matrix containing cerium ions has been proposed as a wound-dressing material. Low concentrations of CeCl_3_ stimulate the proliferation of cardiac fibroblasts in Wistar rats.

Thus, nanoceria containing Ce^3+^ ions in a cellulose matrix can act not only as a vector for delivering highly toxic drugs, including 5FU, but also can provide both synergistic and independent cytotoxicity as well as other types of activity (antioxidant, antibacterial, etc.). Bacterial cellulose–nanoceria composites showed good properties for medical use [34,35,37,39,41]. The synthesis of nanoceria in a bacterial cellulose matrix with uniformly controlled nanoceria distribution in the polymer matrix was previously studied [39]. Despite numerous advantages of bacterial cellulose–nanoceria composites, they are difficult be used for preparing a gel and a film-forming system. The use of gelling cellulose derivatives enables the creation of hydrophilic gels and polymeric films with nanoceria and nanoceria modified with 5FU. Hydrophilic hydroxyethyl cellulose (HEC) and hydroxypropylmethyl cellulose (HPMC) are excellent non-toxic and non-allergic polymers used as film-forming agents and drug carriers in thin polymer films and gels.

In the present work, we proposed the synthesis and studied the properties of nanoceria as a carrier for 5-fluorouracil in a matrix of hydroxyethyl cellulose and hydroxymethylpropyl cellulose for topical dosage form preparation. We studied the following: (i) physicochemical properties and cytotoxicity of nanoceria-modified 5FU; (ii) properties of nanoceria–cellulose derivatives; (iii) design of dosage forms such as gels and polymer films; (iv) permeability of 5FU in gel and polymer films.

## 2. Results

### 2.1. Synthesis of Nanoceria in an HEC and HPMC Matrix

To precipitate CeO_2_ nanoclusters in the HEC and HPMC matrices, we used meglumine, which forms complexes of Ce^3+^ ions with amines and makes it possible to precipitate nanoceria in the polymer matrix. Nanoceria gel dispersions obtained by precipitation with meglumine enable the consistency of HPMC gels and the quality of films with HPMC to be improved. In addition, amines form a salt complex with 5-fluorouracil (5FU) and do not allow 5-fluorouracil to be salted out during storage.

**Figure 1:**
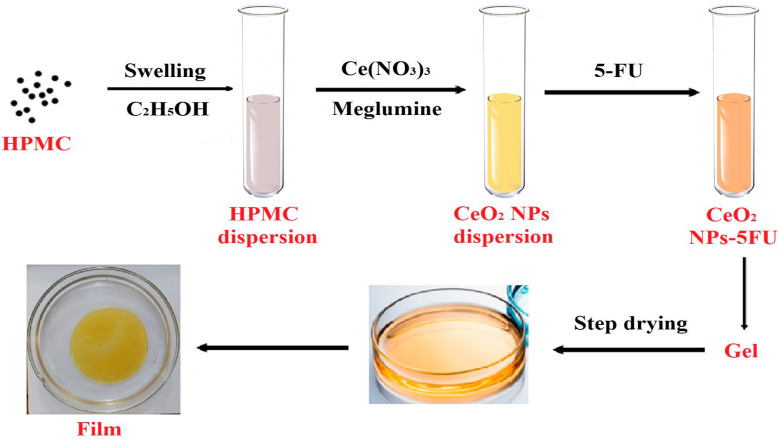
The schematic process of the synthesis of nanoceria modified by 5FU in the HPMC matrix.

The nanoceria (CeO_2_NPs) in the HEC and HPMC matrices were synthesized by treating aqueous (HEC) or alcoholic (HPMC) polymer dispersions, initially with an aqueous or alcoholic solution of cerium nitrate, and then with a meglumine solution until the dispersions acquired a stable yellow color with a slight brown tint. Figure 1 shows a schematic illustration of CeO_2_NPs synthesis in the HPMC matrix.

A dispersion of nanoceria in an HEC-containing medium was used as an aqueous base for preparing gels with 5-fluorouracil. The conditions for obtaining HEC-CeO_2_NPs-5FU hydrogels are given in the experimental section. An alcohol dispersion of nanoceria in an HPMC-containing dispersion was used as a base to obtain polymer films. The films included triethyl citrate as a plasticizer, and Kolliphor and triethanolamine were added as transcutants. The initial concentration of the cerium salt for preparing the HEC-CeO_2_NPs aqueous hydrogels was 0.2% (4.6 mM), which corresponds to a nanoceria concentration in the gel of 4.6 mM. Polymer films of HPMC-CeO_2_NPs were prepared using an alcohol dispersion of HPMC with a cerium salt concentration of 0.33%, which corresponds to 7.6 mM of nanoceria. 

**Figure 2:**
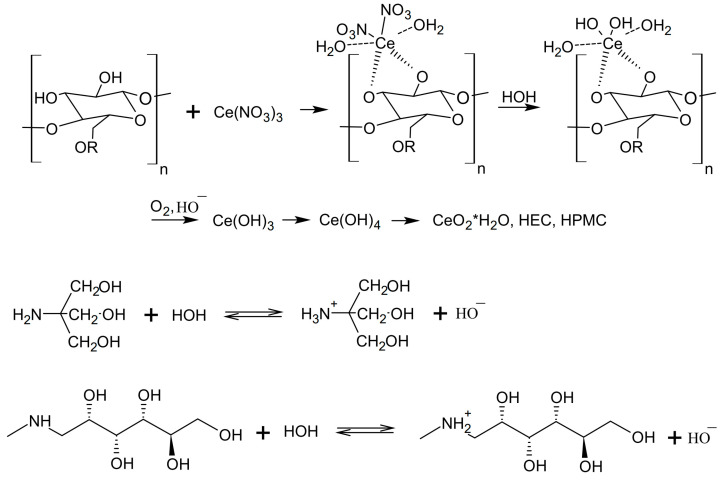
The general scheme of CeO_2_NPs synthesis in the HEC or HPMC matrix.

The general scheme of the reaction is shown in Figure 2.

The alkaline medium (pH > 9.5) is created by meglumine, under the influence of which nanoceria nanoclusters are formed in the polymer matrix.

### 2.2. Physicochemical Properties of Nanoceria Synthesized in a Matrix of HEC or HPMC as Gels and Films

**Figure 3:**
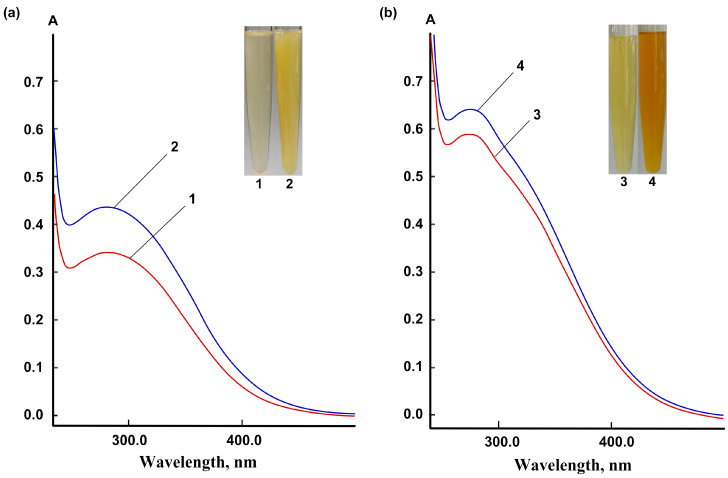
UV spectra of nanoceria dispersions: (**a**) aqueous HEC-CeO_2_NPs (33.3 mg of gel in 1 mL) and (**b**) alcohol HPMC-CeO_2_NPs (20.0 mg of dispersion in 1 mL). Curves 1 and 3—gel formation at 20 °C; curves 2 and 4—dispersions were additionally heated at 60 °C for 3 min.

A band with λ_max_ = 280 nm was observed in the UV spectra of nanoceria in aqueous dispersions of HEC-CeO_2_NPs, which reflects the appearance of an exciton typical of nanoceria (Figure 3). For the complete reaction to proceed according to the scheme shown in Figure 2, the final HEC-CeO_2_NPs dispersions were heated at 60 °C for 3 min. The alcohol dispersions of HPMC-CeO_2_NPs intended for preparing polymer films were dried stepwise with an increase in temperature until the reaction of nanoparticle formation proceeded to completion.

A black-violet color appeared in the HPMC-CeO_2_NPs-5FU and HEC-CeO_2_NPs-5FU gels under UV light, which is typical of CeO_2_NPs compared with the gels of HPMC and HEC alone (Figure S1). 

**Figure 4:**
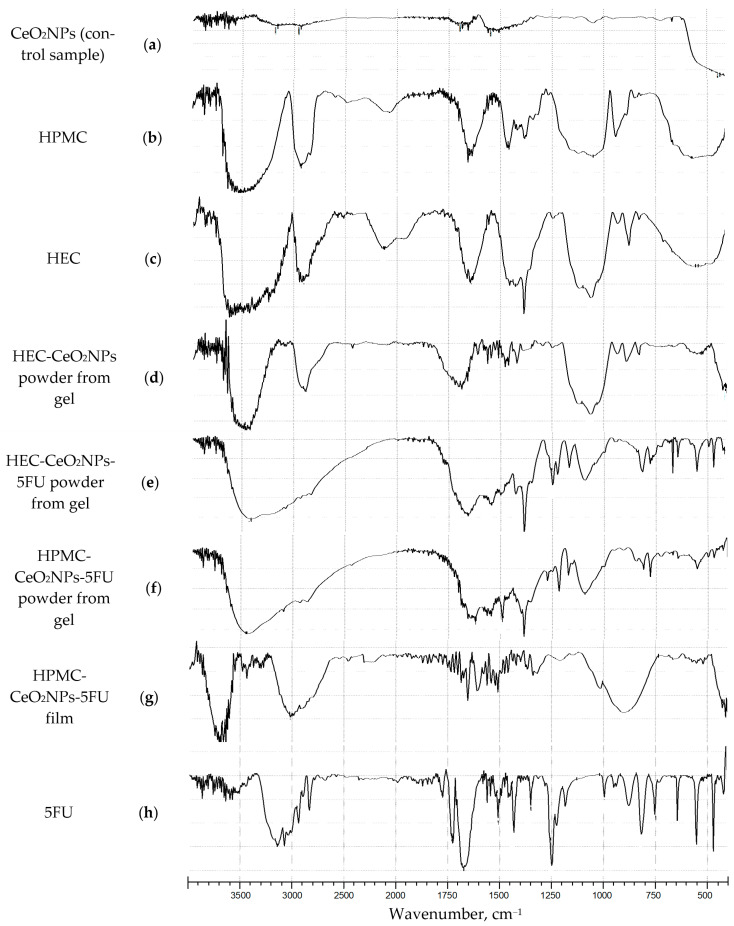
FTIR spectra of a control sample CeO_2_NPs (**a**), HPMC (**b**), HEC (**c**), HEC-CeO_2_NPs powder from gel (**d**), HEC-CeO_2_NPs-5FU powder from gel (**e**), HPMC-CeO_2_NPs-5FU powder from gel (**f**), Film HPMC-CeO_2_NPs-5FU (**g**), 5FU (**h**).

Polymer films and the solid phase obtained from gels were studied by FTIR. The bands of Ce-O stretching vibrations (υ 435–410 cm^−1^) were observed in the FTIR spectra of the films. Similar bands characterized the control sample of nanoceria, as well as the bands of stretching vibrations of -CH, -CH_2_, -CH_3_ (3012–2832 cm^−1^), C-O (1093–1020 cm^−1^) and OH groups (3679–3413 cm^−1^) belonging to the polysaccharide groups of HEC and HPMC (Figure 4, Table 1).

The presence of 5FU was assessed by the band of C-F stretching vibrations (1249–1247 cm^−1^) as well as C=O stretching vibrations in the lactam cycle (1729 and 1638 cm^−1^). In all the films containing 5FU (HEC-CeO_2_NPs-5FU powder from gel, HPMC-CeO_2_NPs-5FU powder from gel, HPMC-CeO_2_NPs-5FU film), C-F and C=O stretching vibration bands were observed.

To study the morphology of HPMC-CeO_2_NPs, HPMC-CeO_2_NPs-5FU polymer films, HEC-CeO_2_NPs and HEC-CeO_2_NPs-5FU, we carried out SEM, EDX and AFM studies. 

**Figure 5:**
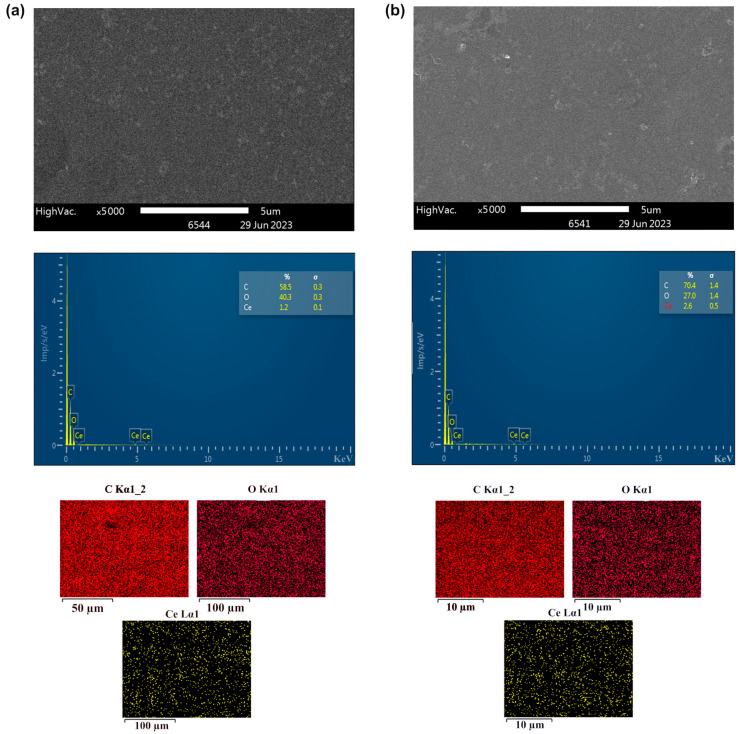
SEM images and EDX spectra of HPMC-CeO_2_NPs films (**a**) and HPMC-CeO_2_NPs-5FU films (**b**).

**Figure 6:**
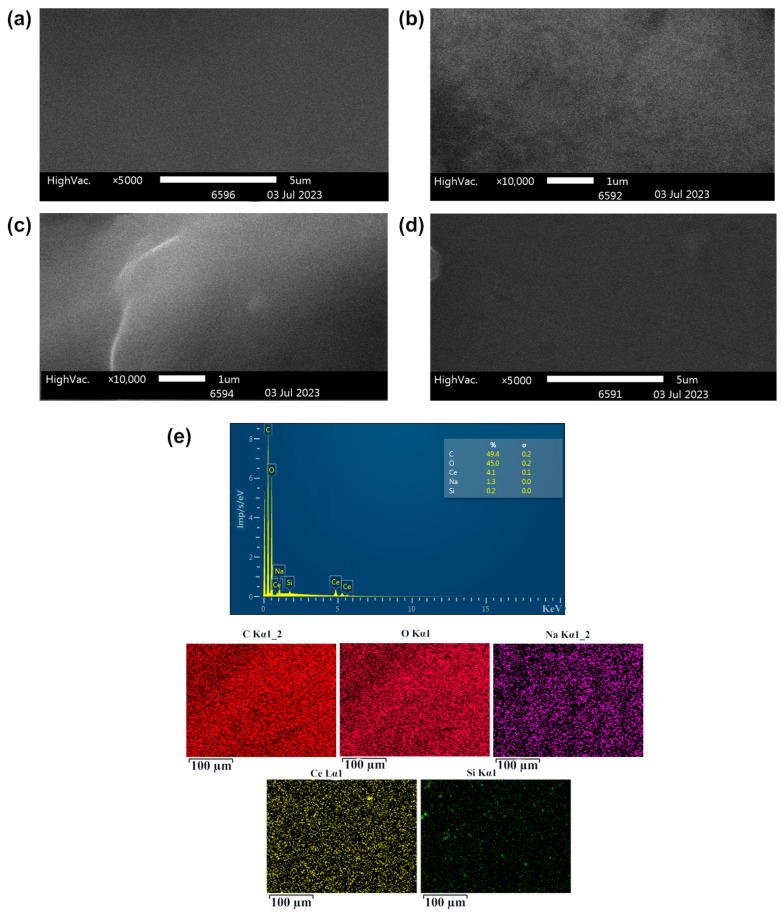
SEM images (**a**–**d**) and EDX spectrum (**e**) of HEC-CeO_2_NPs films (**a**,**c**) and HEC-CeO_2_NPs-5FU films (**b**,**d**) obtained from gels by vacuum drying.

Based on the EDX analysis data, the distribution of cerium in HPMC-CeO_2_NPs and HPMC-CeO_2_NPs-5FU polymer films was found to be uniform, while it was possible to obtain films with a cerium concentration 1.0–3.0% (Figure 5 and Figure 6). To assess the homogeneity of the cerium distribution in the HEC-CeO_2_NPs hydrogel, we obtained the films by vacuum drying.

**Figure 7:**
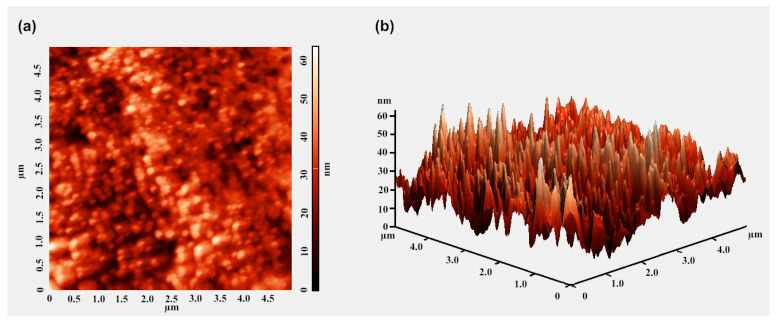
AFM image of the HPMC-CeO_2_NPs-5FU film on a silicon substrate: top view (**a**) and 3D mode (**b**).

To study the topology of the resulting HPMC-CeO_2_NPs-5FU film, it was formed directly on a silicon substrate by pouring a dilute alcohol gel of HPMC-CeO_2_NPs-5FU. An AFM image in the semi-contact mode revealed the granular structure of the nanoparticles coated with a thick polysaccharide layer (Figure 7).

**Figure 8:**
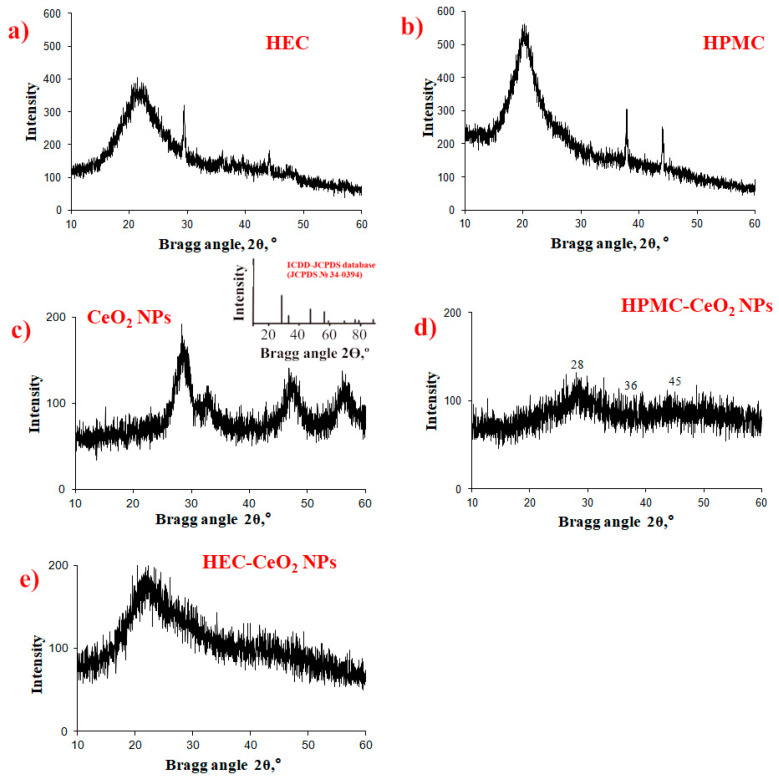
Powder diffraction patterns of HEC (**a**), HPMC (**b**), CeO_2_NPs (**c**), HPMC-CeO_2_NPs-5FU (**d**), HEC-CeO_2_NPs-5FU (**e**). Reflections at 2θ = 37° and 44° refer to the cuvette material.

Figure 8 shows the powder X-ray diffraction (PXRD) data on the CeO_2_NPs, HEC, HPMC and the samples of HPMC-CeO2NPs and HEC-CeO_2_NPs. The structure of the control sample of cerium oxide nanoparticles is cubic of the fluorite type (Figure 8c), which corresponds to powder X-ray cerium (IV) oxide diffraction patterns in the ICDD-JCPDS database (JCPDS No. 34–0394). Weak crystallinity was observed in HEC and HPMC polysaccharides (Bragg angle ~22°, Figure 8a,b). The reflections characteristic of the standard sample of nanoceria are very weak. The absence of a CeO_2_ diffraction maxima on Figure 8d,e is probably due to the shielding effect of the matrix.

**Figure 9:**
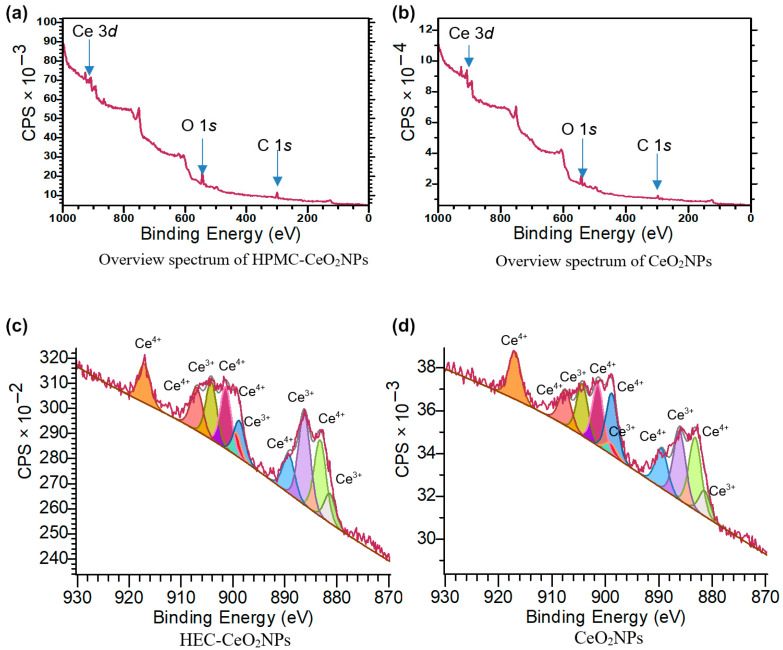
Overview spectra for samples: HPMC-CeO_2_NPs (**a**), control CeO_2_NPs (**b**). Deconvolution of the multiplet 3d-peak of cerium with the background left for the HPMC-CeO_2_NPs samples (**c**) and control CeO_2_NPs (**d**).

The stoichiometry and different oxidation states of cerium in HPMC-CeO_2_NPs samples were estimated by their 3d X-ray photoelectron spectra (3d-XPS). For these experiments, we synthesized nanoparticles by precipitation in an alcoholic 0.1% HPMC solution under the action of meglumine at 60 °C. The results were compared with a control sample of CeO_2_NPs. Figure 9 shows an overview spectrum used to estimate the intensity of the lines and to calibrate the bond energy along the C 1s line.

The deconvolution of the Ce 3d photoelectron lines is significantly complicated by the presence of multiplet splitting due to the spin–orbit interaction of unpaired electrons in the 3d shell. For deconvolution, symmetric lines were used, which are a superposition of the Gauss and Lorentz functions with a percentage ratio of 3/1 for each, respectively. Figure 9 shows different types of spectra for CeO_2_NPs and HPMC-CeO_2_NPs, with and without a background calculation of the concentration of the oxide states Ce^3+^ and Ce^4+^. After the deconvolution, it is possible to determine the percentage of the Ce^3+^ and Ce^4+^ oxidation states for the two samples from the total intensities (Table 2).

The high concentration of Ce^3+^ (30–40%) in CeO_2_NPs in the composition of the HPMC–CeO_2_NPs may reflect the antioxidant, antibacterial and anticancer properties of nanoceria.

### 2.3. The Permeability of 5-Fluorouracil from Gels and Polymer Films through the Acetyl Cellulose Membrane

**Figure 10:**
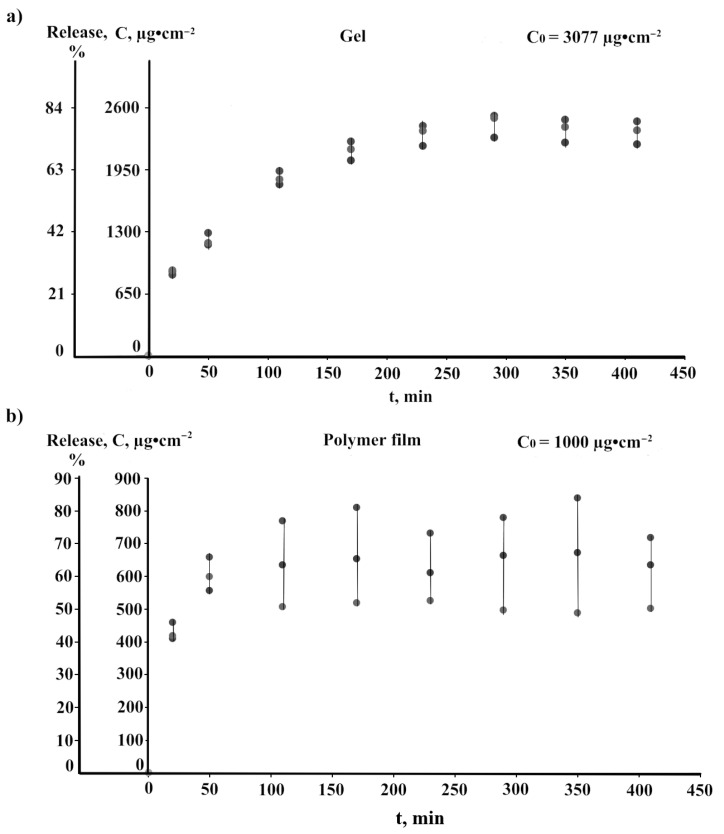
Time dependence of 5FU permeability through the acetyl cellulose membrane from a gel (HEC-CeO_2_NPs-5FU) (**a**) and a polymer film (HPMC-CeO_2_NPs-5FU) (**b**) (*n* = 3). The values of C_0_ = 3077 μg∙cm^−2^ for gel and C_0_ = 1000 μg∙cm^−2^ for a polymer film were correspondingly taken as 100%.

We simulated the dynamics of 5FU penetration on a model of acetyl cellulose membrane using a vertical Franz cell. Figure 10 shows the time dependence of 5FU permeability for the HEC-CeO_2_NPs-5FU gel (Figure 10a) and the HPMC-CeO_2_NPs-5FU film (Figure 10b). An experimental HEC-CeO_2_NPs-5FU gel with an initial 5FU concentration equal to 3077 μg∙cm^−2^ released 75–80% 5FU in 4.0 h. An experimental HEC-CeO_2_NPs-5FU film with an initial 5FU concentration equal to 1000 μg∙cm^−2^ on the membrane released 62–75% 5FU in 1.5 h.

### 2.4. Cytotoxicity Assessment of Nanoceria and Nanoceria Modified by 5FU in Vitro

The qualitative evaluation of the effects of nanoceria on NCTC clone 929 cultures revealed that the nanoparticles actively penetrate into the cells and accumulate mainly in the cytoplasm rather than at the cytoplasmic membrane border (Figure S2). The application of nanoceria at a concentration of 10 μg∙mL^−1^ caused significant cytotoxicity on NCTC clone 929 cells (grade 3), whereas the use of 50 μg∙mL^−1^ and higher caused an acute cytotoxic effect on cell morphology (grade 4) (Figure S2). 

The quantitative evaluation of cell viability by the MTT assay reinforced the qualitative analysis results (Table 3). The use of nanoceria at a concentration of 10 μg∙mL^−1^ did not reduce the number of viable and metabolically active cells in cultures. On the other hand, the application of nanoceria at concentrations exceeding 50 μg∙mL^−1^ caused a significant cytotoxic effect on NCTC clone 929 cells (Table 3). According to the cytotoxicity grading scale, the application of nanoceria at concentrations of 50 μg∙mL^−1^ and 100 μg∙mL^−1^ decreased the number of viable and metabolically active cells to significantly cytotoxic values (grade 3). In the cultures treated with nanoceria at concentrations of 200 μg∙mL^−1^, 400 μg∙mL^−1^ and 600 μg∙mL^−1^, the number of viable cells was at or below the upper limit of the 50% threshold (grade 4) (Figure S1, Table 3).

The cytotoxicity of nanoceria in dispersions (100 μg∙mL^−1^) on tumor cells was also studied for nanoceria modified by 5FU (100 μg of nanoceria contains 6 nmol of 5FU).

**Figure 11:**
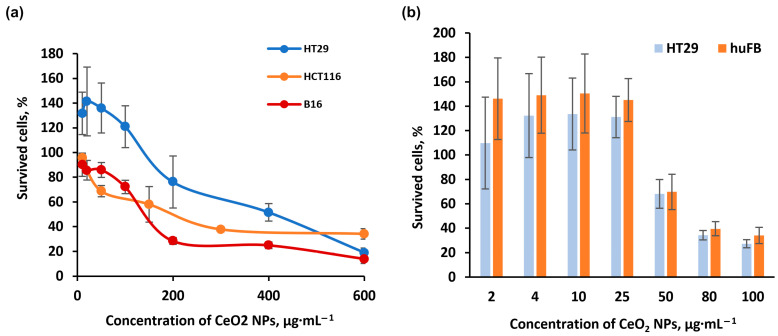
Cytotoxicity assessment of nanoceria 72 h after incubation with normal and cancer cell lines. (**a**) MTT-curve for human colorectal cancer cells (HCT116 and HT29) and murine melanoma B16 cell cultures. (**b**) Diagrams of cell viability for HT29 human colorectal cancer cells and huFB dermal fibroblasts using low concentrations of nanoceria. Statistically significant difference from the control, *p* < 0.05, Kruskal–Wallis test (*n* = 10).

The cellular response to nanoceria showed opposite results, depending on the cell type. Cytotoxic effects of nanoceria were characteristic for the human colorectal cancer HCT116 cell line and murine melanoma B16 cells. The corresponding IC_50_ values were established as 219.0 ± 45.6 μg∙mL^−1^ and 189.5 ± 25.7 μg∙mL^−1^, respectively (Figure 11a). However, the application of nanoceria at low doses up to 25 μg∙mL^−1^ caused an increase in proliferation activity in the human colorectal cancer HT29 cells and human skin fibroblasts (huFB) (Figure 11b).

**Figure 12:**
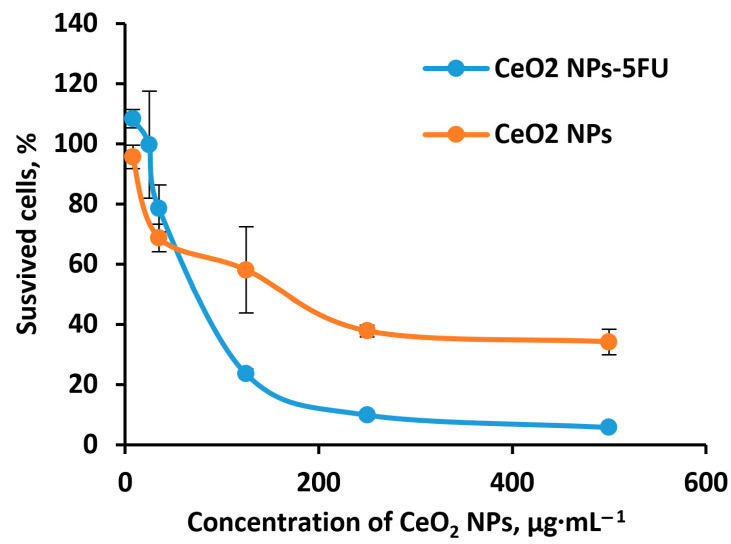
Cytotoxicity assessment of CeO_2_NPs and CeO_2_NPs-5FU 72 h after incubation with HCT116 human colorectal cancer cells. Statistically significant deference from the control, *p* < 0.05, Kruskal–Wallis test (*n* = 10).

Next, we performed the comparative analysis of the effects of pure CeO_2_NPs and CeO_2_NPs-5FU on the HCT116 human colorectal cancer cells. The IC_50_ values for pure CeO_2_NPs and CeO_2_NPs-5FU were 219.0 ± 45.6 μg∙mL^−1^ and 89.2 ± 14.0 μg∙mL^−1^, respectively (Figure 12). The concentration of 5FU in the combination of CeO_2_NPs-5FU was 6.0 µM. In the absence of CeO_2_NPs, the IC_50_ of 5FU for HCT116 was established as 8.7 ± 2.4 µM.

## 3. Discussion

The aggregation and agglomeration of cerium oxide nanoparticles, the relationship between the particle shape and the stability and stoichiometry of Ce^3+^/Ce^4+^ in cerium oxide, as well as the effect of these factors on biological activity, remain debatable. Based on studies by the authors [46,47,48], an increase in the Ce^3+^ percentage in the HPMC-CeO_2_NPs sample, obtained indirectly, characterizes a decrease in the particle size compared with the control sample. The authors believe that spherical particles are characterized by a high catalytic activity, and that it is desirable to use CeO_2-x_ nano powders consisting of particles that do not have well-defined faceting. The cerium oxide particles we obtained (HEC-CeO_2_NPs and HPMC-CeO_2_NPs) were uniformly distributed in the polymer material (SEM, EDX), had the form of spherical particles on the film (AFM), contained 41% Ce^3+^ (XPS) and did not exhibit cytotoxicity on normal cells; thus, they can be good candidates for APIs and dosage forms.

In our work, the effect of the formation of uniformly distributed nanoceria in the gel and in the film is firstly due to meglumine, which results in the smallest sizes of nanoceria as well as other amines, for example, hexamethylenetetramine [47,48]. Secondly, Ce^3+^ ions in cerium nitrate are able to form complexes with the hydroxyl groups of HEC and HPMC, similarly to the interaction with the hydroxyl groups of other polysaccharides (dextrans, maltodextrins, etc.) [49]. In the weakly alkaline environment of amines, HEC and HPMC units acting as ligands do not prevent the hydrolysis and oxidation of Ce^3+^ to Ce^4+^. In this case, Ce(OH)_4_ and CeO_2_ are successively formed (scheme in Figure 2). With an excess of ligands (HEC and HPMC units), the formation of CeO_2_ nanoparticles begins only after the oxidation stage (UV control by the exciton band, Figure 3). It can be assumed that the mechanism of the formation of CeO_2_ nanoparticles under the action of meglumine is close to the formation of nanoceria upon oxidation and precipitation with urea or hexamethylenetetramine [47,48]. The advantage of using these amines as a precipitant is the ability to control the supersaturation degree and avoid the occurrence of local concentration gradients arising during the usual mixing of solutions of a cerium salt and a precipitant. The use of compounds approved for medicinal use, such as cerium nitrate (an antibacterial and anti-burn ingredient) and meglumine (an excipient), as precursors reduces the requirements for the further purification of gels, since toxic substances requiring removal are absent.

Nanoceria isolated in a polymer shell by repeated reprecipitation with acetone and ethanol can be an independent ingredient in other dosage forms. Such particles loaded with 5-fluorouracil (HEC-CeO_2_NPs-5FU and HPMC-CeO_2_NPs-5FU) will be of interest not only for the treatment of skin diseases (psoriasis, dermatitis, vitiligo, etc.) but also in colorectal cancer treatment.

The interest in 5FU treatment in practical medicine has not decreased over time with the development of new dosage forms and new drug delivery systems. Polymeric films and polymeric gels offer many advantages in terms of effective drug delivery and improved patient comfort. In this work, we proposed and studied a polymeric gel and film containing 5FU and nanoceria that have potential skin-healing properties. The release and permeability of 5FU through the acetyl cellulose membrane as a skin model from the gel (75–80% for 4 h) and from the film (62–75% for 1.5 h) characterize the HEC-CeO_2_NPs-5FU gel and HPMC-CeO_2_NPs-5FU film as effective dosage forms suitable for treating skin diseases.

It can be assumed that the differences in cellular responses in cancer cell lines are related to their genomic differences. Thus, HCT116 and B16 cells express the wild-type version of the anti-oncogene TP53, which regulates apoptosis, while HT29 cells have a mutation in TP53 (mutation R273H). This mutated TP53 results in apoptosis inhibition, which can explain the absence of a therapeutic effect in HT29 cell line since the induction of apoptosis was postulated as one of the main mechanisms of action for nanoceria [50].

Thus, a combination of nanoceria and 5FU enables the dose of nanoceria to be reduced more than 2-fold and the 5FU dose to be reduced 1.5-fold without a loss of therapeutic efficacy . Therefore, the application of nanoceria as an anticancer agent requires a selective approach since a dependence of the inhibitory effect on the cancer type was revealed. In addition, the activating effect of low doses of nanoceria on dermal fibroblasts opens up the prospect of using nanoceria as a wound-healing agent.

## 4. Materials and Methods

### 4.1. Materials

5-Fluorouracil (5FU) was purchased from the Chemische Fabric Berg, GmbH, Germany. In the study, we used hydroxyethyl cellulose (Natrosol^®^ 250 HHR, Ashland industries, Rotterdam, The Netherlands), hydroxypropylmethyl cellulose (60HD50, Jinan Maissen New Material Co., Ltd., Jiaxing, China), triethyl citrate (Citrofol AI Pharma, Jungbunzlauer Ladenburg GmbH, Germany), ethanol (95.0% purity, Vekos, Nizhny Novgorod, Russia), cerium nitrate hexahydrate (Khimkraft LLC, Kaliningrad, Russia), aqueous ammonia 25% (SIGMATEK LLC, Khimki, Russia), cellulose acetate membrane (d–0.45 μm, LenReaktiv, Saint Petersburg, Russia), meglumine (99.9% purity, LenReaktiv, Saint Petersburg, Russia), sodium chloride (99.9% purity, LenReaktiv, Saint Petersburg, Russia), potassium phosphate trihydrate (99.0% purity, LenReaktiv, Saint Petersburg, Russia), and dipotassium phosphate trihydrate (99.0% purity, LenReaktiv, Saint Petersburg, Russia).

### 4.2. Synthesis of CeO_2_NPs as a Control Sample

Nanoceria were synthesized according to Karakoti [51], with minor modifications. Briefly, cerium nitrate hexahydrate (2.175 g, 5 mmol) was dissolved in 100 mL of a water–ethylene glycol solution (30:70) and placed in a flask. An aqueous ammonium hydroxide solution (25%) was added dropwise very slowly with vigorous stirring at 60 °C until a pH of 10–11 was attained. After vigorous stirring for three h at 60 °C, a yellow-beige precipitate formed, which was then separated by centrifugation and washed.

### 4.3. Nanoceria-5FU Gel Preparations

A quantity of 0.8 g of HEC was dissolved in 35 mL of distilled water with constant stirring for 40 min and then allowed to swell fully until the polymer dissolved. A quantity of 0.1 g of cerium nitrate hexahydrate was dissolved in another flask in 5 mL of distilled water, and then it was added to the HEC dispersion. Meglumine (0.87 g) and 5-fluorouracil (0.5 g) were dissolved in 10 mL of distilled water under ultrasound treatment for 2 min. At the last stage, a solution of 5-fluorouracil with meglumine was added to the polymer dispersion with constant stirring. Initially, the appearance of a bright golden color was observed, which gradually turned into a brown color. The gel produced is stable for several months. The general composition of the gel is presented in Table 4.

### 4.4. Dermal Polymer Film Preparations

(a) A quantity of 1.0 g of HPMC was dispersed in 17 mL of ethanol (96%) until a transparent homogeneous dispersion without inclusions formed. (b) Cerium nitrate hexahydrate (0.1 g) in ethanol (3 mL) was added to the HPMC dispersion with constant stirring, and then 0.1 g of Kolliphor^®^ P188 was added to the HPMC dispersion. (c) Meglumine (0.33 g), triethanolamine (1.0 g) and 5-fluorouracil (0.1 g) were dissolved in 6 mL of ethanol (96%) under ultrasound treatment for 1 min. (d) Then, the final solution was added to the HPMC solution containing cerium nitrate followed by adding 1.0 g of triethyl citrate as well. A homogeneous gel without bubbles was obtained. Films were formed from the resulting gel in a Petri dish or on a glass substrate. The films were dried in the following stages: air drying for 40 min, then at 60 °C for 60 min, after which they were kept for 120 min while gradually reducing the temperature to 25 °C.

Table 4 shows the general composition of the dermal polymer films.

### 4.5. FTIR Analysis

FTIR spectra were obtained in the 400–4000 cm^−1^ range by an IR Prestige-21 FTIR spectrometer (Shimadzu, Kyoto, Japan). The resolution was 0.5 cm^−1^, and the number of scans was 45.

### 4.6. UV Analysis

UV spectra were obtained by the UV-1800 spectrophotometer (Shimadzu, Kyoto, Japan).

### 4.7. Powder X-ray Diffraction Analysis

Powder X-ray diffraction patterns were recorded by the Shimadzu X-ray diffractometer XRD-6000 (Shimadzu, Kyoto, Japan) at 295 (2) K with Cu Kα radiation (λ = 0.15418 nm) using Bragg–Brentano reflection geometry.

### 4.8. SEM and EDXMA Studies

The samples were visualized by scanning electron microscopy (SEM) using a JSM-IT300LV (JEOL, Tokyo, Japan) microscope with an electron beam diameter of about 5 nm and a probe current below 0.5 nA (operating voltage 20 kV). The surface topography of the powders was studied using low-energy secondary electrons and backscattered electrons. The elemental composition of the powders was studied using X-ray microprobe analysis (XRM) with an X-MaxN 20 detector (Oxford Instruments, Oxfordshire, England).

### 4.9. Atomic Force Microscopy

The morphology of HPMC-CeO_2_NPS-5FU particles deposited from an alcohol solution onto silicon was studied by atomic force microscopy in the semi-contact mode on a Solver P47 (NT-MDT) instrument (Zelenograd, Russia) at room temperature.

### 4.10. Permeability Study

The permeability of 5FU and CeO_2_NPs was studied using a Franz cell (Figure S3) with an acceptor chamber volume of 4.35 mL and 12.65 mL, respectively. An acetyl cellulose membrane (d–0.45 μm) with an area of 1.3 cm^2^ was used as a model of the stratum corneum.

UV-visible spectrophotometry was used to evaluate 5FU permeability from the HEC-CeO_2_NPs-5FU gel and the HPMC-CeO_2_NPs-5FU film. The acceptor solution (PBS at pH 7.4) was analyzed using UV spectra at λ_max_ = 266 nm, and the amount of drug was determined by using a calibration curve generated from known concentrations of 5FU to calculate the percentage of drug released according to Equation (1):

(1) $$Drug\;release\left(\%\right)=\frac{The\;amount\;of\;drug\;released\;at\;time}{The\;total\;amount\;of\;drug\;loaded\;onto\;the\;sample}$$

All the experiments were carried out in triplicate, and the data were expressed as mean ± standard deviation.

### 4.11. Cytotoxicity Assessment in Vitro

#### 4.11.1. Cell Cultures

HCT116, HT29 and B16 cell lines were obtained from the Cell Culture Collection of the D.I. Ivanovsky Institute of Virology Division, N.F. Gamaleya National Research Center of Epidemiology and Microbiology of the Ministry of Health of the Russian Federation (Moscow, Russia). HuFB cells were isolated from healthy donor skin at the Privolzhsky Research Medical University. The established cell line NCTC clone 929 (C3H/An mouse, subcutaneous connective tissue, clone of L. line) was kindly provided by Prof. D.V. Krysko, Cell Death Investigation and Therapy Laboratory, Department of Human Structure and Repair, Ghent University (Ghent, Belgium).

In vitro experiments were performed using the following established cell lines: NCTC clone 929 (C3H/An mouse, subcutaneous connective tissue, clone of L. line), two cell lines of human colorectal cancer (HT29 and HCT116), murine melanoma B16 cells and normal human skin fibroblasts (huFB).

The cells were cultured in DMEM medium (PanEco, Moscow, Russia) supplemented with 10% fetal bovine serum (FBS, Biosera, Cholet, France), 2 mM glutamine and 1% penicillin/streptomycin (PanEco, Russia). At the end of the exponential growth period, the cells were treated with a versene–trypsin solution (3:1) and then reseeded at a multiplicity of sieving of 1:3–1:6 and an approximate cell density of1.0–3.0 × 10^4^ cells∙mL^−1^. The cells were passaged three times a week for cancer cells and NCTC clone 929 cells and once a week for human fibroblasts. The cell viability was maintained in a Binder C150 CO_2_ incubator (BINDER GmbH, Germany) at 37 °C and humidified atmosphere containing 5% CO_2_. All the experiments were performed after the third passage.

#### 4.11.2. Qualitative Cytotoxicity Analysis

NCTC clone 929 cells were seeded in 96-well culture plates at 7×10^3^ cells per well and grown overnight. After cell attachment and subconfluence control, the conditioned culture medium was replaced with complete growth medium containing CeO_2_NPs at concentrations ranging from 10 μg∙mL^−1^ to 600 μg∙mL^−1^. The experimental solutions were prepared in 100% dimethyl sulfoxide (DMSO) under sterile culture box conditions. To minimize the risk of contamination, the stock solutions were passed through a syringe filter with a membrane pore size of 0.2 µm (Techno Plastic Products, Switzerland). The NCTC clone 929 cell cultures, in which the culture medium was replaced with a medium containing DMSO in a ratio of 1:1, served as a positive control. The negative control group of NCTC clone 929 cell cultures was subjected to conditioned medium replacement with complete growth medium in a ratio of 1:1. 

Qualitative cytotoxicity was assessed 24 h after CeO_2_NPs application according to the ISO 10993-5:2009, with some modifications [52,53,54]. At least 10 fields of view were analyzed for each culture. According to the cytotoxicity score, >50% of dead cells in a culture corresponded to acute cytotoxicity (grade 4), 30–50% indicated significant cytotoxicity (grade 3), 20–30% indicated moderate cytotoxicity (grade 2), 10–20% indicated light cytotoxicity (grade 1), and 0–10% was considered non-cytotoxic (grade 0).

#### 4.11.3. Quantitative Cell Viability Assay

A quantitative cell viability analysis was performed using the MTT test [52,53,55].

NCTC clone 929 cells were grown and treated with CeO_2_NPs for 24 h as described above (see Section 4.11.2). For other examined culture cell lines, the cells were seeded in 96-well plates (5 × 10^3^ cells per well) and grown overnight. Then the cells were exposed to 8–600 µg∙mL^−1^ solution of CeO_2_NPs-5FU and incubated for 72 h. 

After the incubation period, the culture medium was replaced with a serum-free medium containing the MTT reagent (5 mg∙mL^−1^, Alfa Aesar, UK). After 2 h, the medium was removed, and the formed formazan crystals were dissolved in DMSO. The optical density (*E*) of the solution was measured at 570 nm and 620 nm wavelengths using a Synergy Mxmulti-mode microplate reader (BioTek Instruments, Winooski, VT, USA). The proportion of viable cells (*N*v) was calculated according to Formula (2):

*N*v = *E*_experimental_/*E*_control_ ×100% (2)

Triplicate wells were set up in each plate and three independent experiments were performed to determine the IC_50_ concentration.

#### 4.11.4. Statistical Analysis

The data were statistically processed using GraphPad Prism v.9.3.1.471 software (San Diego, CA, USA) and the nonparametric Kruskal–Wallis test. The hypothesis of normal distribution was tested using the Shapiro–Wilk test. The differences were considered statistically significant if the *p* value was less than 0.05.

## 5. Conclusions

In summary, 5FU-loaded gelling cellulose derivatives (HEC-CeO_2_NPs-5FU and HPMC-CeO_2_NPs-5FU) were developed to study their physicochemical properties, drug permeability and potential effects on normal and tumor cells. CeO_2_NPs were immobilized in cellulose-derivative matrices by co-precipitation with meglumine, then 5FU was loaded into HEC-CeO_2_NPs-5FU and HPMC-CeO_2_NPs-5FU gels. FTIR, XPS and EDX analyses showed the successful use of HEC-CeO_2_NPs-5FU and HPMC-CeO_2_NPs-5FU as potential active pharmaceutical ingredients (API). CeO_2_NPs in these substances have a higher concentration of Ce^3+^ (41%) compared with pure CeO_2_NPs without matrix (34%), which is good for antioxidant and antibacterial properties.

The studies on the permanent cell line NCTC clone 929 showed that cerium oxide nanoparticles actively penetrate into the cytoplasm of cells and have a slight cytotoxic effect associated with changes in cell morphology, even at 10 μg∙mL^−1^. The use of nanoparticles protected with polymers at 10 μg∙mL^−1^ did not significantly affect the morphology and metabolic activity of the NCTC clone 929 cells.

It was shown that when using the HCT116 human colorectal cancer cells, the combination of nanoceria and 5FU enabled the dose of nanoceria to be reduced by more than 2-fold without losing its therapeutic efficacy, while the 5FU dose was reduced by 1.5-fold.

We developed novel compositions of the HEC-CeO_2_NPs-5FU hydrogel and the HPMC-CeO_2_NPs-5FU film. The optimum 5FU concentration was equal to 1% for the HEC-CeO_2_NPs-5FU hydrogel and 0.33% for the HPMC-CeO_2_NPs-5FU film. The SEM and AFM images revealed that cellulose derivatives displayed a role as a matrix for nanoceria with a uniform distribution of nanoceria (EDX). The gel and film exhibited colloid stability. The 5FU permeability from the HEC-CeO_2_NPs-5FU gel and the HPMC-CeO_2_NPs-5FU film was estimated to be 75–80% for 4–5 h and 62–75% for 1.5–2 h, respectively. This fact demonstrates the efficiency of the proposed dosage forms and would enable the use of various dosage forms depending on the disease.

Therefore, HEC-CeO_2_NPs-5FU and HPMC-CeO_2_NPs-5FU could be potential drug carrier candidates due to their physicochemical and anticancer activities, and the HEC-CeO_2_NPs-5FU gel and HPMC-CeO_2_NPs-5FU film could be effective topical dosage forms.

## Figures and Tables

**Table 1:** Data (υ, cm^−1^) of FTIR spectra of the studied samples.

Sample	3600–3200 (-OH, -NH)	3100–2700 (-CH, -CH_2_, -CH_3_)	1753–1650 (C=O in 5FU) 1248–1249 (C-F)	1600–1300 (C-O, NO^3−^)	570–425 (Ce-O)
CeO_2_NPs	-	3161	-	1535, 1071, 1020	435
HPMC	3445	2931	-	1053	-
HEC	3526	2906	-	1062	-
HEC-CeO_2_NPs powder from gel	3462	2882	-	1060	410
HEC-CeO_2_NPs-5FU powder from gel	3413	-	1659, 1543, 1247	1092	420
HPMC-CeO_2_NPs-5FU powder from gel	3407	2832	1653, 1550, 1247	1093	419
HPMC-CeO_2_NPs-5FU film	3679	3012	1728, 1673, 1249	1020–895	424
5FU	3141	2932	1729, 1638, 1248	-	-

**Table 2:** Results of 3d-XPS spectra of HPMC-CeO_2_NPs and control CeO_2_NPs.

Sample	Ce^3+^, %	Ce^4+^, %
HPMC-CeO_2_NPs	41	59
CeO_2_NPs	34	66

**Table 3:** MTT analysis of NCTC clone 929 cell viability 24 h after incubation with nanoceria (M ± SD) ^1^.

	Concentration, μg∙mL^−1^						# IC_50_, μg∙mL^−1^
Viable cells, % of negative control (taken as 100%)	10	50	100	200	400	600	190.1 [128,1; 282,3]
	94.5 ± 3.6	54.6 ± 5.3 *	50.2 ± 5.1 *	47.6 ± 5.8 *	43.2 ± 2.4 *	44.8 ± 1.8 *	

^1^ Positive control corresponded to 5.0 ± 0.4 * % (of negative control); * statistically significant differences relative to the negative control, *p* < 0.05, Kruskal–Wallis test (*n* = 6); # a log-normal distribution model was used to calculate the IC_50_; mean values and boundaries of the 95% confidence interval are indicated.

**Table 4:** Optimal compositions of a gel and a dermal polymer film with nanoceria-5FU.

Dosage Form	Component	Weight, g
Gel	HEC	0.80
	Cerium (III) nitrate hexahydrate	0.10
	Meglumine	0.87
	5-fluorouracil	0.50
	Water	up to 50.00
Dermal polymer film	HPMC	1.00
	Cerium (III) nitrate hexahydrate	0.10
	Meglumine	0.33
	5-fluorouracil	0.10
	Kolliphor^®^ P188	0.10
	Triethanolamine	1.00
	Triethyl citrate	1.00
	Ethanol (96%)	26.00

## Data Availability

Data is contained within the article and supplementary material.

## Supplementary Materials

The following supporting information can be downloaded at: https://www.mdpi.com/article/10.3390/ph16081082/s1, Figure S1: Photo images of HPMC-CeO_2_NPs-5FU and HPMC gels under daylight (a) and UV light (b). Photo images of HEC-CeO_2_NPs-5FU and HEC gels under daylight (c) and UV light (d); Figure S2: Cytotoxicity evaluation of nanoceria on NCTC clone 929 cells line 24 h after incubation. Note–scale bar 100 µm; Figure S3: Schematic design of a vertical Franz diffusion cell. Cell volume was equal to 12.65 mL and 4.35 mL.
