# Supplementary material for: Design and Study of Nanoceria Modified by 5-Fluorouracil for Gel and Polymer Dermal Film Preparation
Source: Pharmaceuticals (Basel). 2023 Jul 29;16(8):1082. doi: 10.3390/ph16081082 (PMC10458209; doi:10.3390/ph16081082)
Supplement: Supplementary file 1 [file pharmaceuticals-16-01082-s001.zip › pharmaceuticals-2522639-supplementary.pdf]

**(a)** **(b)** (λ = 365 nm) **(c)** **(d)** (λ = 365 nm)

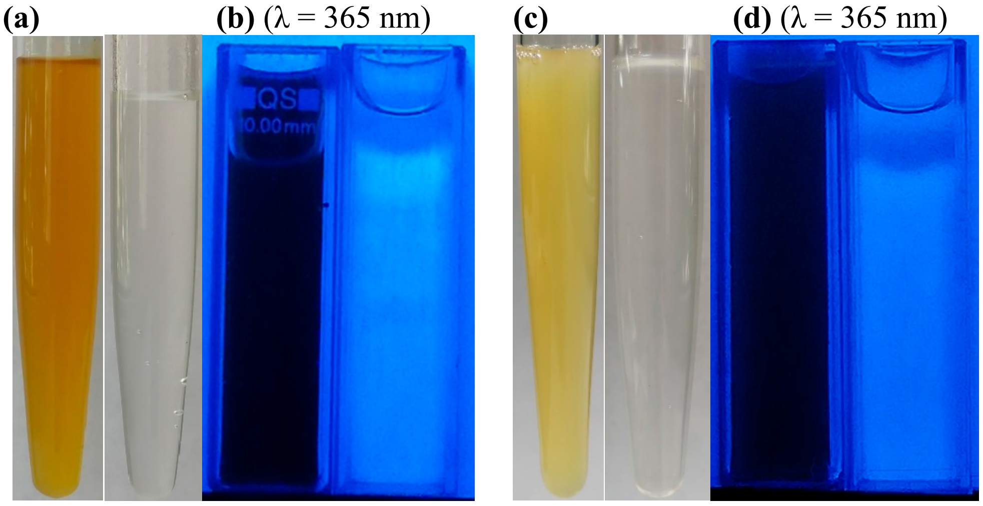

HPMC-$CeO_2$NPs-5FU gels HEC-$CeO_2$NPs-5FU gels

**Figure S1.** Photo images of HPMC-$CeO_2$NPs-5FU and HPMC gels under daylight (a) and UV light (b). Photo images of HEC-$CeO_2$NPs-5FU and HEC gels under daylight (c) and UV light (d).

| **Concentration, $\mu g \cdot mL^{-1}$ (cytotoxicity grade scale)** | **Representative photomicrographs of cultures** | |
|---|---|---|
| | **10x** | **20x** |
| Negative control (grade 0) | 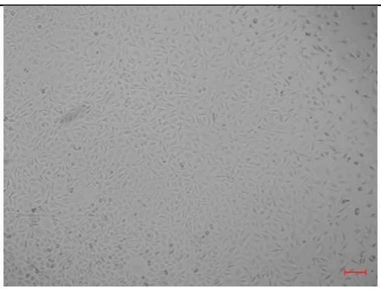 | 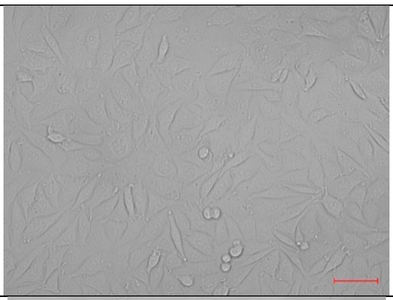 |
| Positive control (grade 4) | 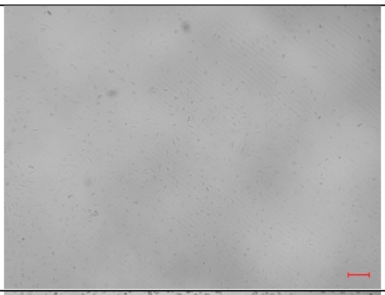 | 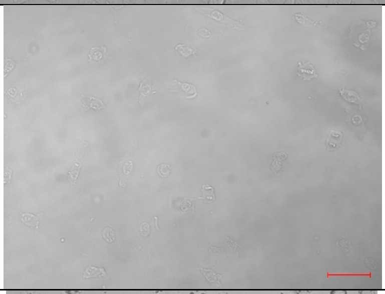 |
| 10.0 (grade 3) | 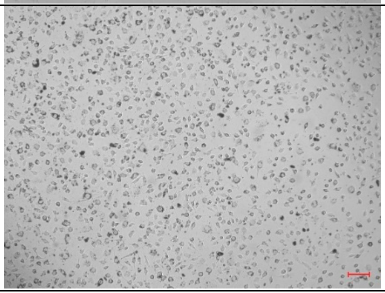 | 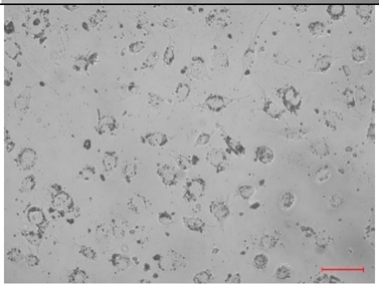 |

| | | |
|---|---|---|
| 50.0<br>(grade 4) | 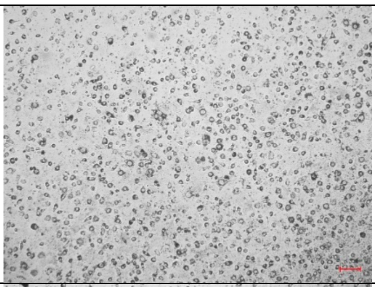 | 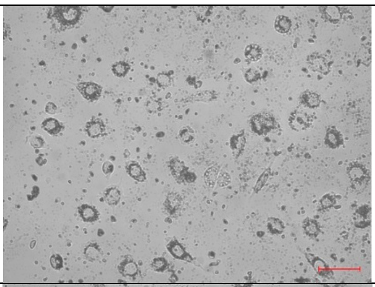 |
| 100.0<br>(grade 4) | 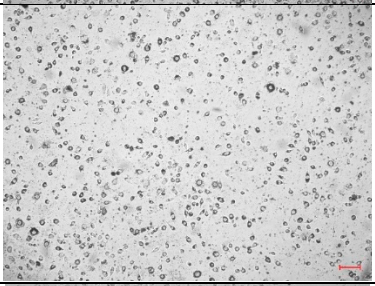 | 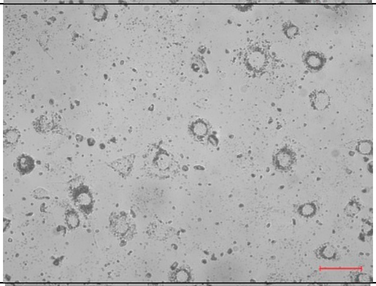 |
| 200.0<br>(grade 4) | 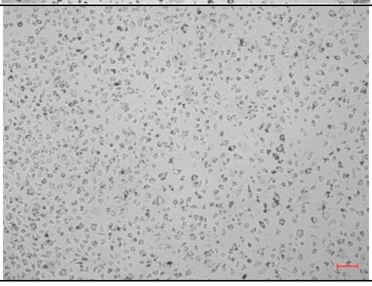 | 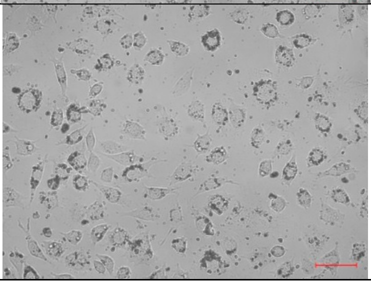 |

**Figure S2.** Cytotoxicity evaluation of nanoceria on NCTC clone 929 cells line 24 h after incubation. Note – scale bar 100 µm.

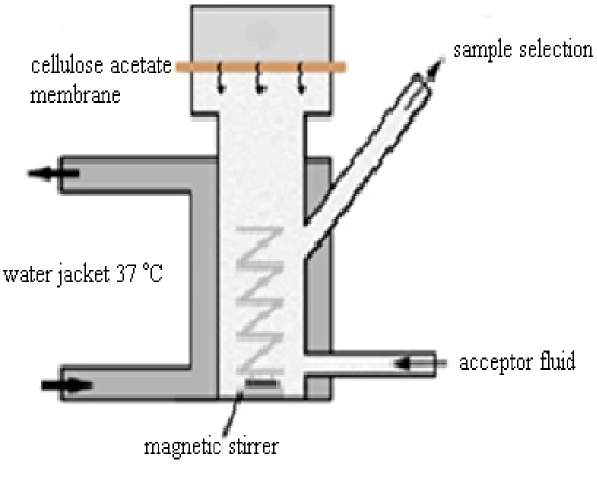


**Figure S3.** Schematic design of vertical Franz diffusion cell. Cell volume was equal to 12.65 mL and 4.35 mL.
